# Evaluation of envelope domain III-based single chimeric tetravalent antigen and monovalent antigen mixtures for the detection of anti-dengue antibodies in human sera

**DOI:** 10.1186/1471-2334-11-64

**Published:** 2011-03-15

**Authors:** Gaurav Batra, Satish K Nemani, Poornima Tyagi, Sathyamangalam Swaminathan, Navin Khanna

**Affiliations:** 1 Recombinant Gene Products Group, International Centre for Genetic Engineering & Biotechnology, New Delhi, India

## Abstract

**Background:**

Flavivirus cross-reactive antibodies in human sera interfere with the definitive identification of dengue virus (DENV) infections especially in areas with multiple co-circulating flaviviruses. Use of DENV envelope domain-III (EDIII) can partially resolve the problem. This study has examined the effect of (i) incorporating the EDIIIs of four DENV serotypes into a single chimeric antigen, and (ii) immobilizing the antigen through specific interaction on the sensitivity and specificity of anti-DENV antibody detection.

**Methods:**

A sera panel (n = 164) was assembled and characterized using commercial kits for infection by DENV and a host of other pathogens. Anti-DENV antibodies of both IgM and IgG classes in this panel were detected in indirect ELISAs using a mixture of monovalent EDIIIs, a chimeric EDIII-based tetravalent antigen, EDIII-T, and a biotinylated version of the latter as coating antigens. The sensitivity and specificity of these assays were compared to those obtained using the PanBio Dengue IgG/IgM ELISAs.

**Results:**

The performance of dengue IgG and IgM indirect ELISAs, using either a physical mixture of four EDIIIs or the single chimeric EDIII-T antigen, were comparable. Coating of a biotinylated version of the tetravalent antigen on streptavidin plates enhanced sensitivity without compromising specificity.

**Conclusions:**

The incorporation of the EDIIIs of the four DENV serotypes into a single chimeric antigen did not adversely affect assay outcome in indirect ELISAs. Oriented, rather than random, immobilization of the tetravalent antigen enhanced sensitivity of detection of anti-DENV antibodies with retention of 100% specificity.

## Background

Dengue viruses (DENV), of which there are four serotypes (DENV-1,-2,-3 and -4), are mosquito-borne flaviviruses of the *Flaviviridae *family, which also includes other members, such as yellow fever virus, Japanese encephalitis virus, West Nile virus and tick-borne encephalitis virus (TBEV) [1]. Currently, there is no vaccine to prevent or a drug to treat DENV infection, which poses a public health threat to nearly half the global population [2]. In this scenario, the availability of reliable diagnostic tools assumes great importance in clinical management, surveillance and outbreak investigations. As DENVs share antigenic similarities with other flaviviruses and tend to co-circulate with some of them in many endemic areas, the unambiguous detection of anti-DENV antibodies using currently available commercial kits, which use mixtures of inactivated virus preparations or recombinant envelope proteins for antibody detection, is often not possible [2].

Efforts to eliminate the problem of cross-reactivity have begun to focus on the utility of DENV envelope protein domain III (EDIII), as a diagnostic intermediate of high specificity [3-5]. As this domain contains both serotype-specific as well as DENV complex-specific epitopes [6], it is necessary to utilize EDIIIs of all four DENV serotypes to detect anti-DENV antibodies. Recently, we designed a single recombinant chimeric tetravalent antigen, EDIII-T, by linking the EDIIIs of the four DENV serotypes [5]. However, the sensitivity of this antigen in detecting anti-DENV antibodies in enzyme linked immunosorbent assays (ELISA) was not as high as that of the reference assays. This may have been the result of unavailability of some of the epitopes, arising either from the incorporation of the EDIIIs into a tetravalent design, or, due to random adsorption of the EDIII-T antigen on the polystyrene surface during the performance of ELISAs. To address these issues we have expressed and purified four monovalent DENV EDIII antigens [7,8] and a biotinylated version of EDIII-T antigen (b-EDIII-T) [8], for oriented immobilization on a streptavidin-coated surface.

The major aims of this study were to (i) compare the performance of single EDIII-T antigen with a physical mixture of monovalent EDIIIs corresponding to the four DENV serotypes; and, (ii) evaluate if oriented immobilization of the tetravalent antigen influences the sensitivity of detection of both IgG and IgM classes of anti-DENV antibodies, in indirect ELISA. We report here the outcome of a parallel evaluation of a physical mixture of EDIIIs, EDIII-T and b-EDIII-T as diagnostic antigens in ELISAs for the detection of anti-DENV antibodies in human sera.

## Methods

### Study design

A panel of 164 sera obtained from both dengue-endemic and non-endemic regions was pre-screened for evidence of infection by DENV, TBEV and a variety of non-flavivirus pathogens including Chikungunya virus, *Plasmodium*, *Leptospira*, and *Salmonella *using commercially available kits. This panel was used in indirect ELISAs to evaluate the performance of a mixture of monovalent EDIIIs, EDIII-T and b-EDIII-T as diagnostic reagents in detecting anti-DENV antibodies.

### Materials

Goat anti-human IgG (γ-chain specific)-horseradish peroxidase (HRP), and goat anti-human IgM (μ-chain specific)-HRP conjugates were purchased from Calbiochem (La Jolla, CA, USA). HRP substrate 3, 3', 5, 5'-Tetramethylbenzidine (TMB) was from Sigma-Aldrich (St. Louis, MO, USA). Maxisorp polystyrene ELISA plates and immobilizer streptavidin ELISA plates were from Nunc-Thermo fisher scientific (Roskilde, Denmark). Dengue IgG/IgM capture ELISA test was from PanBio Diagnostics (Brisbane, Australia) and TBEV IgG/IgM ELISA was from Virion/Serion GmbH (Würzburg, Germany). The Advantage Pan Malaria Card test for *Plasmodium *LDH antigen and the Lepto IgM micro-ELISA test for *Leptospira interrogans *antibodies were from J. Mitra & Company Ltd. (New Delhi, India). The Typhi-Dot test for *Salmonella typhi *antibodies was from MBDr Diagnostics Sdn Bhd (Selangor, Malaysia) and the Insight Chik V rapid test for chikungunya antibodies was from Tulip Diagnostics Ltd. (Goa, India).

### Human sera

The human sera samples (n = 164) used in this study were from hospitals in India and Finland. Sera were drawn in the early symptomatic phase after informed written consent of the patients. Ethical permissions for the collection of blood samples were obtained from the ethical committees of the respective hospitals. All sera were pre-screened for anti-DENV antibodies using the PanBio Dengue IgG/IgM capture ELISA test. Eighty samples scored positive and 84 scored negative for DENV IgG whereas 81 samples scored positive and 83 scored negative for DENV IgM antibodies. Of the DENV IgM^-^/IgG^- ^sera samples (n = 72), there was evidence of infection by TBEV (n = 18), *Plasmodium *(n = 6), *Leptospira interrogans *(n = 5), *Salmonella typhi *(n = 6) and Chikungunya virus (n = 4).

### Recombinant EDIII antigens

The monovalent EDIIIs antigens used in this study, corresponding to the four DENV serotypes, each contain 121 amino acid (aa) residues spanning DENV envelope aa296-aa416. Their expression and purification have been reported earlier [7,8]. The design of the tetravalent antigen EDIII-T containing the EDIIIs of the four DENV serotypes, linked in tandem, its expression and purification have been described previously [5]. To produce the biotinylated tetravalent antigen b-EDIII-T, we used an *in vivo *method wherein the biotinyl moiety is added to a specific site enzymatically [8]. The chemical method which tends to add the biotinyl moiety in a random fashion to available acceptor groups is not quite suitable for achieving uniform and oriented binding on streptavidin coated surfaces. In order to carry out site-specific enzymatic biotinylation of the EDIII-T antigen *in vivo*, a biotin acceptor peptide (BAP) was cloned in frame with the amino terminus of the EDIII-T antigen and co-expressed with biotin ligase in *E. coli*. The engineered BAP moiety of the recombinant tetravalent antigen serves as the substrate for biotin ligase resulting in the production of *in vivo *biotinylated tetravalent antigen, b-EDIII-T [8].

### IgG and IgM indirect ELISA

Polystyrene ELISA plate wells were coated either with 100 μl of EDIII-T (3 μg/ml), b-EDIII-T (3 μg/ml) or a mixture of four EDIII antigens (0.75 μg/ml of each EDIII) in 0.1 M carbonate buffer, pH 9.5, and incubated overnight at 4°C. The wells were blocked with 200 μl of blocking solution (4% skim milk in 1x PBS, pH 7.2) at 37°C for 2 hours and washed 3x with 1x PBS, pH 7.2/1% Tween 20/0.5 M KCl. For DENV specific IgG detection in sera, washed wells were incubated with 100 μl patient serum diluted 1:100 in IgG assay diluent (1x PBS, pH 8.0/2.5% skimmed milk/2% polyvinyl pyrrolidone/10% goat serum/0.1% BSA/1% Tween 20/0.5 M KCl) at 37°C, for 45 minutes. Plates were washed and incubated with 100 μl of anti-human IgG-HRP conjugate (diluted 1:10,000 in IgG assay diluent) at 37°C, for 30 minutes. Next, wells were incubated with 100 μl of TMB soluble substrate at 37°C, for 15 minutes. The reaction was stopped by adding 100 μl of 1 M H_2_SO_4 _and the absorbance read at 450 nm. The IgM indirect ELISA was essentially similar, except for the following differences. Sera were diluted 1:80 in IgM assay diluent (IgG assay diluents lacking goat serum and KCl) and pre-incubated with Serion Rf-Absorbent as recommended by the manufacturer (Virion/Serion GmbH, Würzburg, Germany) to remove IgG antibodies before using in the assay. Bound anti-DENV IgM antibodies were detected using anti-human IgM-HRP conjugate (diluted 1:2,500 in IgM assay diluent). In some experiments, the sera were tested on streptavidin ELISA plates coated with 100 μl of b-EDIII-T antigen (250 ng/ml, in 1x PBS, pH 7.0/0.5% Tween 20/1% skim milk) for 2 hours, at 30°C, with shaking, at 200 rpm. Wells were washed and used for IgG and IgM indirect ELISAs as above. For each of the ELISAs performed, the mean absorbance of the DENV IgM^-^/IgG^- ^serum samples (n = 72) plus three times the standard deviation was designated as the cut-off value (see footnotes to Tables 1 and 2). We used this relatively stringent cut-off to minimize the possibility of picking up false positives. Results were expressed as signal to cut-off (S/Co) ratios. Sera with S/Co ratios ≥ 1.0 were designated as positive and those with ratios < 1 as negative.

**Table 1:** Comparison of EDIII-based monovalent antigen mixture and single tetravalent antigen in anti-DENV antibody detection^*a*^

	IgG (*n = 80*)			IgM (*n = 81*)		
		
Parameter	EDIII Mix	EDIII-T	Mean Ratio of S/Co **values (95% CI)**^b^	EDIII Mix	EDIII-T	Mean Ratio of S/Co **values (95% CI)**^b^
% Sensitivity^*c*^	81.3	82.5	1.22 (1.15-1.29)	59.3	63.0	1.11 (1.06-1.16)

% Specificity^*d*^	100	100	-	100	100	-

^*a*^Assays were done using polystyrene microtiter wells that were coated either with a mixture of four EDIII monovalent antigens (EDIII Mix) or the single tetravalent antigen, EDIII-T. The number of sera analyzed is denoted by *n*.

^*b*^This column represents the mean ratio of S/Co values using EDIII-T to S/Co values using EDIII Mix as the coating antigens; the cut-off values were 0.30 and 0.33 for EDIII mix and EDIII-T, respectively, in the IgG assay, and 0.23 and 0.25, respectively, in the IgM assay. The 95% confidence interval is indicated in parentheses.

^*c*^Percent Sensitivity = (Number of sera that scored positive by our test/Number of sera that scored positive using PanBio ELISAs) × 100

^*d*^Percent Specificity = (Number of sera that scored negative by our test/Number of sera that scored negative using PanBio ELISAs) × 100

**Table 2:** The performance of b-EDIII-T as a capture antigen in the detection of anti-DENV antibodies^*a*^

	IgG (*n = 80*)			IgM (*n = 81*)		
		
Parameter	PS	SA	Mean Ratio of S/Co **values (95% CI)**^b^	PS	SA	Mean Ratio of S/Co **values (95% CI)**^b^
% Sensitivity^*c*^	82.5	91.3	1.32 (1.24-1.39)	61.7	75.3	1.30 (1.23-1.37)

% Specificity^*d*^	100	100	-	100	100	-

^*a*^Assays were done using polystyrene (PS) microtiter wells or streptavidin (SA) coated microtiter wells. The number of sera analyzed is denoted by *n*.

^*b*^This column represents the mean ratio of S/Co values using SA plates to S/Co values using PS plates; the cut-off values were 0.31 and 0.28 for PS and SA, respectively, in the IgG assay, and 0.23 and 0.25, respectively, in the IgM assay. The 95% confidence interval is indicated in parentheses.

^*c*^Percent Sensitivity = (Number of sera that scored positive by our test/Number of sera that scored positive using PanBio ELISAs) × 100

^*d*^Percent Specificity = (Number of sera that scored negative by our test/Number of sera that scored negative using PanBio ELISAs) × 100

### Statistical analyses

To compare the performance of the single tetravalent EDIII-T antigen with that of a mixture of the monovalent EDIII antigens in detecting anti-DENV antibodies, the S/Co values were used to calculate a ratio by dividing the S/Co value obtained using EDIII-T antigen by the S/Co value obtained using the EDIII monovalent antigen mixture. Similarly, the performance of b-EDIII-T in streptavidin-coated ELISA plates versus polystyrene plates was assessed from a ratio of the corresponding S/Co values. The significance of the relative sensitivity was assessed by obtaining the 95% confidence interval (CI) using an alpha value of 0.05, the calculated standard deviation and the sample size. Statistical calculations were performed using MS Excel 2003 software.

## Results

### Comparison of EDIII antigen mixture versus a single chimeric EDIII-T antigen in indirect ELISAs

**Figure 1:**
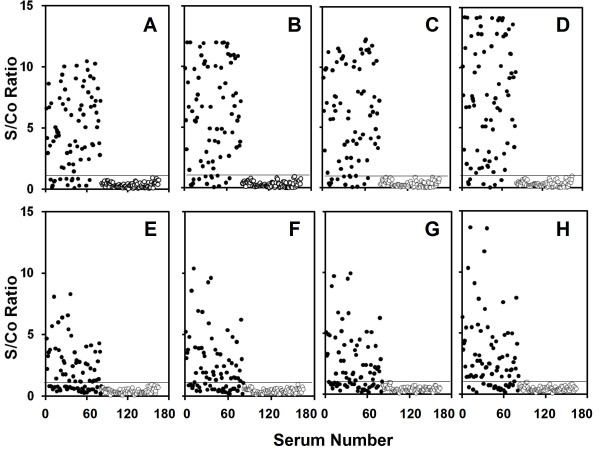
**Comparative analysis of DENV-specific antibodies in patient sera in indirect ELISAs using DENV EDIII-based coating antigens**. Anti-DENV IgG (panels A-D) and IgM (panels E-H) antibodies were detected using a mixture of four monovalent EDIIIs (panels A & E), EDIII-T (panels B & F), or b-EDIII-T (panels C, D, G & H) as the coating antigen. The antigens were coated on either polystyrene (panels A, B, C, E, F & G) or streptavidin (panels D & H) plates. The S/Co values of individual DENV-positive and -negative sera, clustered together, are indicated by filled and unfilled circles, respectively. The horizontal line in each panel indicates the cut-off corresponding to S/Co ratio of 1.

The reactivity of an antigen mixture comprising the EDIIIs of the four DENV serotypes towards anti-DENV antibodies was compared to that of a single tetravalent antigen incorporating all these four EDIIIs in a single molecule [5]. For this purpose indirect IgM and IgG ELISAs were performed using 164 human sera collected from endemic and non-endemic countries which were pre-characterized for the presence or absence of anti-DENV IgM and IgG antibodies using a commercial kit from PanBio Diagnostics. As the cut-off absorbance values for different antigens were different for the same class antibodies (Tables 1 and 2), results are represented as S/Co ratios to interpret the data correctly (Figure 1). The data reveal that, both the sensitivity and specificity of a physical mixture of 4 monovalent EDIII antigens in detecting anti-DENV antibodies are comparable to those obtained using the single EDIII-T antigen. In fact, the EDIII-T antigen resulted in a modest, yet significant increase in sensitivity (Table 1). The S/Co ratios for DENV positive samples tended to be slightly higher in EDIII-T based ELISAs (compare Figure 1A with 1B for IgG and 1E with 1F for IgM).

### Determination of the effect of oriented immobilization of the tetravalent antigen in indirect ELISA

It has been suggested that specific immobilization of antigen on the ELISA plate well can improve the performance of the immunoassay compared to passive coating of antigen [9-11]. To test if this may be true for the detection of anti-DENV antibodies using the tetravalent antigen, we used a biotinylated version of this antigen to achieve specific and oriented immobilization on streptavidin treated ELISA plates. This demonstrated that, indeed, b-EDIII-T coated on streptavidin plates was superior in sensitivity in comparison to passively coated non-biotinylated EDIII-T antigen in detecting IgG as well as IgM antibodies (compare Figure 1B with 1D for IgG; and 1F with 1H for IgM; p < 0.05 in both cases). This enhancement in assay sensitivity was not evident if the b-EDIII-T antigen was passively coated on polystyrene plates (compare Figure 1B with 1C for IgG; and 1F with 1G for IgM). The data in Table 2 show that the use of b-EDIII-T coated on streptavidin can enhance the efficiency of detection of both IgG and IgM anti-DENV antibodies, as evidenced by 30% increase in the mean S/Co values.

## Discussion

This study was undertaken to investigate if the antigenic epitopes on the chimeric tetravalent EDIII-T antigen are available and accessible to anti-DENV antibodies. To this end, the ELISA reactivity of a physical mixture of the 4 EDIII antigens was compared to that of the EDIII-T antigen using a panel of 164 human sera that were pre-screened with a battery of commercially available tests for DENV, TBEV, Chikungunya virus, *Plasmodium*, *Leptospira*, and *Salmonella*. The lower specificity of the commercial Dengue IgG/IgM capture ELISA (PanBio Diagnostics), which served as a reference in this study, is well documented [12]. An assessment of the true sensitivity of the EDIII-based assays described here was precluded due to non-availability of adequate sera for characterization by virus neutralization tests. However, this does not adversely affect the conclusions of this study as it compares the relative performance of the tetravalent EDIII-T antigen against a mixture of monovalent EDIIIs and evaluates the effect of using biotinylated versus non-biotinylated EDIII-T in detecting serum anti-DENV antibodies.

In both IgM and IgG indirect ELISAs the sensitivities manifested a small but significant improvement when the coating antigen was the single tetravalent antigen compared to the physical mixture of four EDIIIs (Table 1). Thus, this analysis showed that the individual EDIII components are well displayed in the EDIII-T antigen. We also found that biotinylation of the tetravalent antigen enhanced the efficiency of anti-DENV antibody detection further, by 30%, when it was immobilized on streptavidin-treated, but not on untreated surface. Further, this enhancement was statistically significant (Table 2). Additionally, the amount of biotinylated antigen (25 ng per well) required was a fraction of that of its non-biotinylated counterpart (300ng/well). This is presumably a reflection of better availability of the b-EDIII-T antigen stemming from its orderly and oriented immobilization through its biotinyl moiety to the streptavidin surface. This is consistent with the notion that passive, random coating on polystyrene surfaces offers only a small fraction of the antigen for interaction with antibodies [10].

Using b-EDIII-T coated on streptavidin surface, the sensitivity of the IgG-ELISAs was ~91% (Table 2). In the IgM assay, sera were pre-cleared with rheumatoid factor absorbents to eliminate possible IgG interference. Yet, sensitivity of IgM detection was consistently lower and the maximum that could be achieved was ~75% (Table 2). The observation that the results of EDIII-T based ELISAs were slightly better than those obtained with the mixture of EDIIIs argues against the possibility that the tetravalent design may have contributed to the lower sensitivity. A more likely possibility is that natural DENV infections generate a complex polyclonal response targeting a whole range of viral epitopes with EDIII-specific antibodies comprising only a small proportion of the total anti-DENV antibody population [13].

A recently reported comparative analysis of several commercial DENV IgM antibody ELISAs has shown that most of these suffer from low specificity, with sera from a large percentage of malaria- and leptospirosis-positive individuals scoring positive for anti-DENV IgM antibodies [12]. To address the issue of false positives, we included in our study a collection of 39 DENV IgM^-^/IgG^- ^sera that were positive for infection by several other pathogens such as *Plasmodium*, *Leptospira*, *Salmonella*, TBEV and Chikungunya virus. Regardless of whether we used EDIII monovalent antigen mixture or either version of the tetravalent antigen, all these 39 sera were scored as negative samples in both IgM and IgG ELISAs (Figure 1). The level of observed specificity may stem from the EDIII epitopes predominantly recognizing the DENV complex, thereby reducing cross reactivity from antibodies elicited by other non-DENV pathogens.

## Conclusions

The multiepitope antigen design coupled with the requirement of very low quantity per assay has potential cost benefit. However, the sensitivity needs to be enhanced further, presumably by the inclusion of additional DENV specific epitopes. The EDIII based ELISAs may be useful in epidemiological surveillance and vaccine efficacy trials.

## List of abbreviations

DENV: Dengue virus; EDIII: Envelope domain III; ELISA: Enzyme-linked immunosorbent assay; HRP: Horseradish peroxisade; TBEV: Tick-borne encephalitis virus; TMB: 3, 3', 5, 5'-Tetramethylbenzidine.

## Competing interests

The authors declare that they have no competing interests.

## Authors' contributions

GB, SKN and PT performed the experiments. SS and NK conceived the study and wrote the manuscript. All authors read and approved the final manuscript.

## Pre-publication history

The pre-publication history for this paper can be accessed here:

http://www.biomedcentral.com/1471-2334/11/64/prepub
